# A new *in-silico *method for determination of helical transmembrane domains based on the *PepLook *scan: application to IL-2Rβ and IL-2Rγc receptor chains

**DOI:** 10.1186/1472-6807-11-26

**Published:** 2011-05-24

**Authors:** Yan Charlois, Laurence Lins, Robert Brasseur

**Affiliations:** 1 Centre de Biophysique Moleculaire Numerique, ULg Gembloux AgroBiotech, 5030 Gembloux, Belgium

## Abstract

**Background:**

Modeling of transmembrane domains (TMDs) requires correct prediction of interfacial residues for in-silico modeling and membrane insertion studies. This implies the defining of a target sequence long enough to contain interfacial residues. However, too long sequences induce artifactual polymorphism: within tested modeling methods, the longer the target sequence, the more variable the secondary structure, as though the procedure were stopped before the end of the calculation (which may in fact be unreachable). Moreover, delimitation of these TMDs can produce variable results with sequence based two-dimensional prediction methods, especially for sequences showing polymorphism. To solve this problem, we developed a new modeling procedure using the *PepLook *method. We scanned the sequences by modeling peptides from the target sequence with a window of 19 residues.

**Results:**

Using sequences whose NMR-structures are already known (GpA, EphA1 and Erb2-HER2), we first determined that the hydrophobic to hydrophilic accessible surface area ratio (ASAr) was the best criterion for delimiting the TMD sequence. The length of the helical structure and the *Impala *method further supported the determination of the TMD limits. This method was applied to the IL-2Rβ and IL-2Rγ TMD sequences of *Homo sapiens, Rattus norvegicus, Mus musculus *and *Bos taurus*.

**Conclusions:**

We succeeded in reducing the variation in the TMD limits to only 2 residues and in gaining structural information.

## Background

IL-2 and IL-15 are two structurally close hematopoietic cytokines, both presenting a functional redundancy and both involved in immunology and inflammatory diseases. IL-2 was implicated in the first metastatic melanoma immunotherapy [1] and is used in adoptive immunotherapy with tumor infiltrating lymphocytes (TILs). Most treatments require high doses of IL-2 [2,3] and induce a systemic toxicity similar to GM-CSF (Granulocyte Macrophage-Colony Stimulating Factor) treatments [4]. These secondary effects restrict the therapy and have led to investigations into other forms of treatment, such as the promising IL-15 therapy.

Transduction receptor chains IL-2Rβ and IL-2Rγc (class I hematopoietic receptors) form an intermediate-affinity complex capable of binding IL-2 and IL-15. Whereas the IL-2Rβ chain is restricted to dimeric receptor for IL-15 and IL-2 [5] or to specific trimeric receptors (IL-2Rβ/IL-2Rγ/IL-2Rα and IL-2Rβ/IL-2Rγ/IL-15Rα), the IL-2Rγc chain is involved in γc-family receptors, that is to say the IL-2, IL-4, IL-7, IL-9, IL-15, IL-21 and GM-CSF receptors[6]. γc-deficit is implicated in immunodeficiency, such as X-linked Severe Combined ImmunoDeficiency (X-SCID)[7].

*Fluorescence resonance energy transfer (FRET) *studies suggest that IL-2Rα, IL-7Rα and IL-15Rα chains are co-localized[8] with IL-2Rβ and γc chains within lipid rafts[9]. IL-2Rα and IL-15Rα chains also appear to co-localize in lipid rafts with MHC (I and II) chains, with ICAM-1 in antigen-presenting cells (APC), and with lymphoma and CD4+ T-cells[10,11]. The majority of these chains are involved in the immune synapse (IS) [10-12] and this supports the implication of IL-2/IL-15 receptors in this IS complex, contributing to their very fast response to *stimuli*.

Due to the therapeutic interest of IL-2 and IL-15 and their activating properties on the IL-2Rβ/IL-2Rγ receptor, we aimed to study the two chains forming this receptor. However, the structure of these chains remains unknown due to the crystallization problems of transmembrane domains (TMDs).

**Figure 1:**
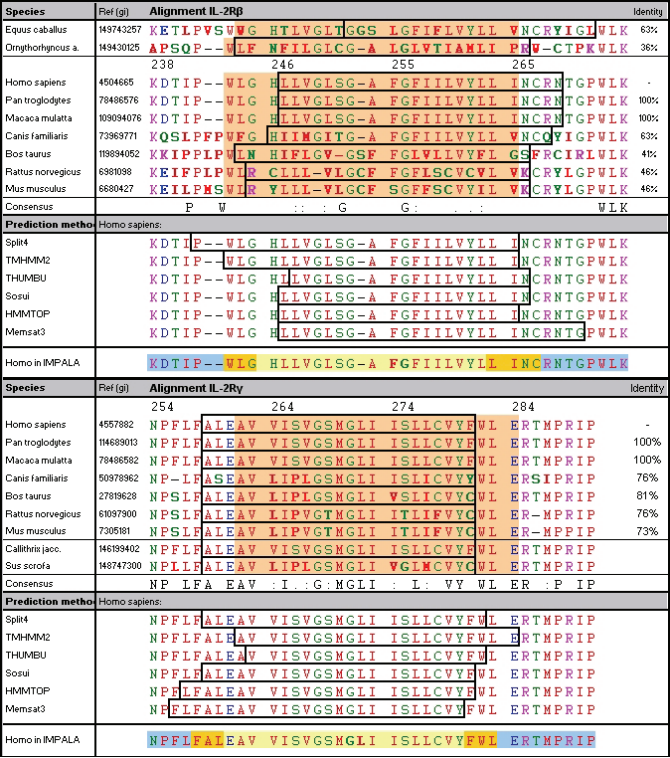
**Inter-species alignment of IL-2R chains and prediction of TMD**. Sequences from different species (gi accession numbers are given) were aligned for the region around the suspected TMD region in IL-2Rβ (left) and in IL-2Rγ (right). Percentages represent the identity of sequences compared to the human sequence of the aligned region (sequence differences in comparison with humans are indicated in bold). The consensus sequence is given: conserved amino acids (a.a.) are indicated by the letter corresponding to these residues. Complete sequences were submitted to TMHMM2 and Sosui web-servers to predict TMD regions: the a.a. included in the TMD according to the *TMHMM2 *prediction are indicated by an orange background, and *Sosui *predictions by boxes. In the second part of the figure, boxes show TMD predictions for the *Homo sapiens *sequence with different methods: *TMHMM2, split4, THUMBU, Sosui, HMMTOP *and *memsat3*. The line "Homo in Impala" shows results from this study: the TMD center (in bold), the predicted interfaces (with orange background) and the juxta-membrane domains (with blue background).

Since no structural models of IL-2Rβ and γc TMDs are available, we needed to model them. We first tried to predict the TMDs using sequences as input via 2D TMD-determination methods (evaluated according to Punta[14] and Zhou[15]) but this led to fluctuations in predicting interfacial residues, according to the species or the method (Figure 1). In the same way, long sequences in 3D structure predicting methods led here to highly flexible TMD models (with a root mean square deviation (RMSD) of between 5 and 19 Å), especially for IL-2Rβ. To resolve this problem, we developed a procedure to define the TMD center by scanning the sequence in different species. This procedure uses the *in-silico PepLook *method[16,17] combined with the *Impala *method for predicting the membrane restraint effect on the structure.

We validated our approach with sequences of proteins with known TMD structures (GpA, EphA1 and Erb2) and succeeded in correctly predicting the TMD limits. We then applied this approach to the IL-2Rβ and IL-2Rγ sequences. Structures of both TMDs were helical, and residues responsible for flexibility or interaction were determined.

## Results

As we were looking for structural and functional data on IL-2Rβ and IL-2Rγc TMDs, we first analyzed the available sequences among different species.

### Multi-species alignment of sequences and transmembrane segment prediction

According to inter-species alignment of IL-2Rβ sequences (Figure 1), identity is low and only two glycines (G253 and G256) around the TMD center are strictly conserved, separated by 2 or 3 a.a., corresponding to either a GxxG (GG3) pattern (for anthropoids and the duck-billed platypus) or a GxxxG (GG4) pattern (for other non-anthropoids). We can observe that the GG3 motif corresponds to a flip of G{CS} in the GG4 pattern, observed in cows, rats and mice, to {CST}G. The horse sequence shows a glycine insertion just before the GG3 pattern. Sequence alignments show conservation of some charged and polar residues at the extremities of the potential TMD.

The shared IL-2Rγc chain is much more conserved than IL-2Rβ for mammals with an identity higher than 70%. A pattern can be identified: F258AxEAVxIxx**G**xM**G**LIxxLxxVYxWLER285-box1 with a potential GG3 pattern and where box1 represents the cytosolic motif involved in interaction with the second messenger JAK-kinase. Although this chain is shared by 7 different receptors and its sequence would therefore be expected to be conserved, we were very surprised to observe a proline to serine substitution just before the v267**G**sM**G**LI273 conserved region, in pigs, cows, rats, mice and dogs, which could induce a kink.

Since we wanted to model the chains for modeling and interaction studies, the first step was to accurately delimit the TMD: we tested TMD predictions for IL-2Rβ and IL-2Rγc sequences in different species using the Split4, TMHMM2, THUMBU, Sosui, HMMTOP and Memsat3 methods[14,15]. 2D-structure prediction methods confirmed that the TMDs of IL-2Rβ and IL-2Rγc are helical, polytopic and mono-spanning. However, TMD predictions differed with a maximal shift of 8 residues, depending on the species and on the prediction method (Figure 1).

In a first approach, we modeled the whole predicted TMD, composed of 27 a.a. for IL-2Rβ (W243-N269) and 26 a.a. for IL-2Rγc (A259-E284), using *PepLook *[16,17] , in a hydrophobic environment or with *Impala *membrane restraints. *PepLook *allows the prediction of the 3D structure and flexibility using the sequence only as input. IL-2Rβ showed no consensus because of the high structural flexibility of the I258-N269 segment models (with a RMSD for the 99 best structures of between 5 and 19 Å; data not shown). IL-2Rβ was assumed to be contained inside the membrane but it cannot be inserted into the membrane using the *Impala *method[18]. IL-2Rγc presents a much reduced flexibility. We noticed that polymorphism seemed to be sequence-length dependent (over 25 residues) for sequences presenting high flexibility such as IL-2Rβ.

### Calibration of the *in-silico *TMD-determination method

**Figure 2:**
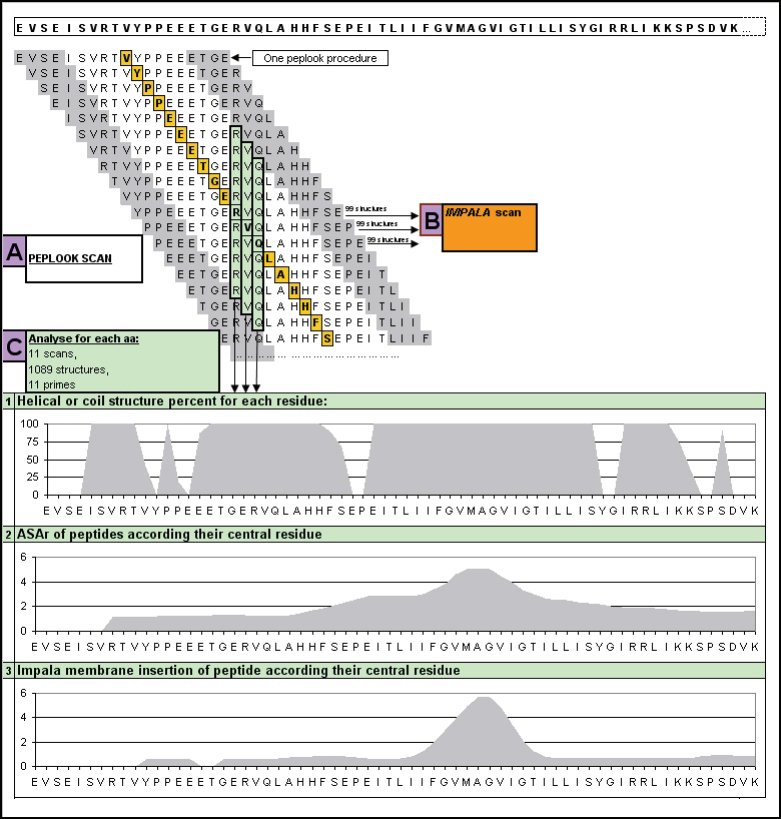
**Illustration of the method applied to the GpA sequence**. **(A) **Scan of peptides with a window of 19 a.a. length, each line represents a *PepLook *run and its 99 structures. **(B) **Scan-peptides were submitted to the *Impala *method for membrane insertion prediction. **(C) **Statistical analysis showing for each residue the average of the percentage of helical structure (%), of the ASAr and of the membrane insertion (distance to the membrane in Å). These data were used to determine TMD residue at the center of the membrane.

We carried out a local prediction by scanning the peptide sequence in a hydrophobic environment with a sliding window, so as to delimit more precisely the TMD limits and flexible domains (Figure 2-A). In order to determine the window length avoiding polymorphism induced by sequence length, we used the TMD sequences from proteins presenting NMR structures with a low RMSD (for the different models from one protein) of between 0.22 and 0.5 Å, with different lengths: we used Gpa (pdb: 1afo), EphA1 (2k1k) and Erb2 (2jwa). We then decided to choose the window length inducing a flexibility of less than the double RMSD observed in NMR models: for this, we determined the window length where the 99 best energetic structures, resulting from the *PepLook *method, were clustered with a RMSD lower than 1 Å. We found that the window length was lower than or equal to 19 a.a.

**Figure 3:**
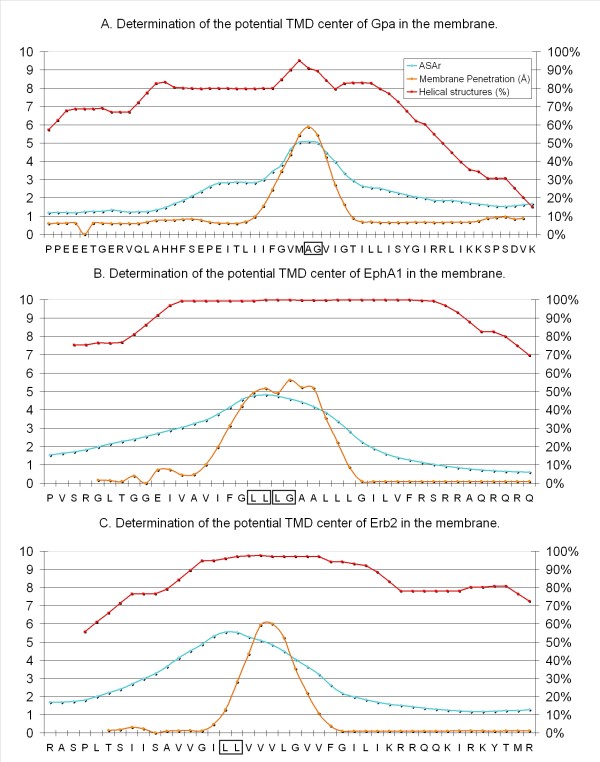
**Determination of the potential TMD center of NMR-structures in the membrane**. According to the statistical results from the *PepLook *scan runs for GpA **(A)**, EphA1 **(B) **and Erb2 **(C)**, we show the average percentage of helical structure in all the structures obtained with a window of 19 a.a. (red line). The blue line represents the average of the ratio of hydrophobic to the hydrophilic accessible surface (ASAr) for 19 a.a. long peptides with a window of 5. The orange line represents the depth of the average penetration into the membrane from 10 to -10 Å of the 19 a.a. long peptides with a window of 3. The amino acid on the × axis represents the center of the considered scan-peptides. TMD centers, determined by the *Impala*[18] and *OPM *methods[20] with NMR structures, are indicated by a box for each monomer considered.

In order to calibrate the strategy, we applied it to Gpa, EphA1 and Erb2, whose NMR structures and TMD central residues are known as a result of using the *Impala *method[18,19] and/or *OPM database *models[20]. We analyzed different parameters for all the peptides generated by the sliding window procedure: the mean of the helical structure with a window of 19 residues, the hydrophobic to hydrophilic accessible surface ratio (ASAr), the average mean force potential (MFP) percentage and the depth of membrane insertion with *Impala*. This means that for each residue, we analyzed 11 scans, generating 1089 structures encompassing the residue of interest. Of all these parameters tested with sequences from known NMR-structures, we found that the best criterion for defining the TMD center was the ASAr mean with the highest value (close to 5) for the central residue compared to a value of around 2 for interfacial amino acids. This was confirmed by the mean of the helical structure, which must be up to 75%. The *Impala *method also confirmed these central residues, except for Erb2, for which we observed a shift of 3 residues (Figure 3-C).

### Structural prediction of the IL-2Rβ TMD

**Figure 4:**
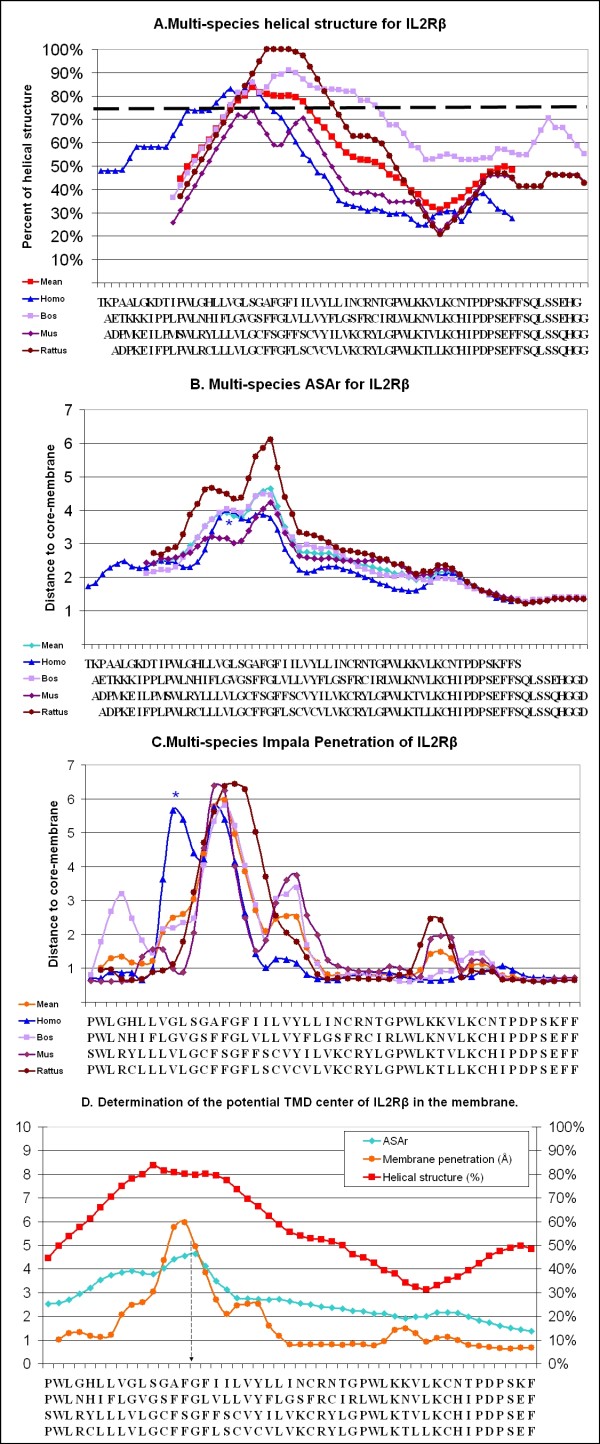
**Determination of the potential TMD center of IL-2Rβ and IL-2Rγ in the membrane**. According to the statistical results from the *PepLook *scan run for the IL-2Rβ **(4) **and the IL-2Rγ **(5), (A) **the average percentage of helical structure in all the structures obtained with a window of 19 a.a. is shown for the different species as follows: *Homo sapiens *(blue), *Bos taurus *(purple), *Mus musculus *(dark purple), and *Rattus norvegicus *(dark red). The amino acid on the × axis represents the center of the considered scan-peptides, according to the species considered. The inter-species mean is shown in red and the acceptable TMD limits are represented by a dashed line at 75%. **(B) **The average ASArs are shown for the 4 species with a window of 5 amino acids; the inter-species mean is shown in blue. **(C) **We show the average depth of the penetration of the 19 a.a. long peptides, with a window of 3, in the membrane from 10 to -10 Å for the 4 species, and the inter-species mean (orange). **(D) **The means of each parameter are shown in the same graph, and the deducible TMD center is indicated by a dashed arrow. (*) Blue stars show the single variations described for *Homo sapiens*.

Following these results, we submitted the IL-2Rβ sequence from 4 different species (*Homo sapiens, Rattus norvegicus, Mus musculus *and *Bos taurus*) to this procedure. The four species have a similar helical structure, ASAr and membrane penetration (Figure 4-ABC): ASAr and the *Impala *scan allowed us to predict that the TMD is centered on F255 and G256, which is consistent with the percentage of helical structure at these positions (close to 80%). However, in *Homo sapiens*, two close maxima were observed for insertion and ASAr (see blue stars in Figure 4-B &4C). The W243 residue of IL-2Rβ seemed to be localized at the extracellular membrane interface and Y262 on the intracellular side, which is consistent with known data regarding TMDs[21]. The scan-peptides presenting the highest RMSD were centered on residues from L260 to L264 (more than 1 Å as against less than 0.1Å for other peptides).

### Structural prediction of the IL-2Rγc TMD

The same procedure was applied to IL-2Rγ sequences: the predicted TMD is centered on G271 and L272 for humans and on homologous residues for other species. Despite an identical result for ASAr in all species, human structures presented a shift of two residues in the *Impala *scan (centered on S269, M270 and G271). This chain structure is less flexible than for IL-2Rβ but peptides centered on residues from F281 to L283 presented a RMSD higher than 0.8Å.

### MFP analysis

**Figure 5:**
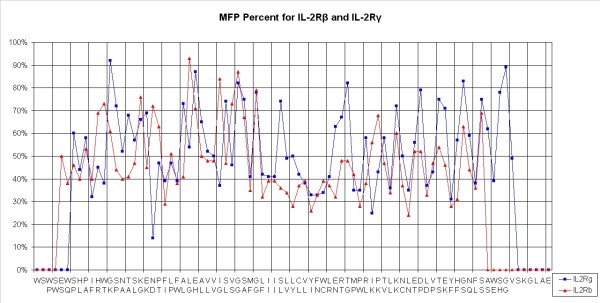
**Determination of the MFP percentage for IL-2Rβ and IL-2Rγ scanned sequences**. After modeling the scan-peptides corresponding to the sequence of both proteins, means of the MFP percentages across the sequence were calculated and are shown (IL-2Rβ in red and IL-2Rγ in blue).

The MFP percentage[22] allows us to predict whether a residue is in a stable structural environment (value higher than 60%) or not (lower than 40%). The structures obtained with our scan method were used to calculate MFP values. Concerning IL-2Rβ, the flexible I258-N269 segment presented an MFP percentage of lower than 40%: such values mean that residues are not stabilized and would therefore need to interact. I241 and Y262 and I265 interfacial residues presented MFP percentages of lower than 30% and, in the center of the TMD, F255 and F257 also had low MFP percentages (Figure 5). For IL-2Rγ, cytoplasmic residues of TMD extremities presented low MFP values as for IL-2Rβ (less than 40%) but for fewer residues: L272-I274 and C278-W282. The interfacial residue N254 presented an extremely low MFP percentage with only 14%.

## Discussion

### Efficiency of the local scan with the *PepLook *procedure

The absence of 3D structures for IL-2Rβ and IL-2Rγ led us to model them *in-silico*. Calculation time for such modeling simulations increases with the number of residues and with the use of supplementary restraints.

We first modeled the whole potential TMD, but this led to a long calculation time and structural flexibility at both membrane interfaces with artifactual loops going back into the membrane; moreover, we found that polymorphism increased with sequence length. Results of sequence-based TMD 2D-predictions did not allow us to delimit TMDs accurately or to reduce the target sequence. In addition, we found no improvement with these 2D-predictions for our target proteins as compared to the use of more basic methods, which include analyzing the hydrophobicity scale or the most frequently present residues at the membrane interface (Trp>Phe>Tyr>Leu>Iso>Cys) or the charged residues usually present at the juxta-membrane interface (Glu and Asp at extracellular side, and Lys and Arg at the cytosolic side), as reviewed by White[21] and Popot[23].

In this paper, we developed an *in silico *method (Figure 2-A) consisting of a local scan with a 19 a.a. window using the *PepLook *method, in order to delimit the TMD more accurately. This scan also allows us to predict other structural properties such as structural polymorphism and potential interaction residues.

We calibrated our method by delimiting precisely intra-membrane regions of mono-spanning receptors using proteins whose NMR structures are known and whose central residues are determined: GpA, EphA1 and Erb2. We analyzed different parameters for all the peptides generated: the helical structure, the ASAr, the average MFP percentage and the depth of membrane insertion with *Impala*. The best criterion for defining the TMD center was found to be ASAr, which presented the highest value for the central residues. The percentage of helical structure and membrane insertion with the *Impala *method provided complementary data to support this result. However, the *Impala *parameter of scanned-peptides showed different results in the case of Erb2.

In order to understand this phenomenon, we split the reference structure of GpA into small peptides from 22 to 33 residues and submitted them to the *Impala *membrane insertion method: the orientation and the penetration of the peptide into the membrane were very variable for small peptides, even for a shift of only one residue (See additional file 1). We also noticed that charged residues were not always sufficient to explain this. So we attribute the different results in determining the central residue of Erb2 to the importance of the residues at the end of the peptides for membrane insertion.

Moreover, the above procedure could be very useful for determining minimal protein sequences that could be used in experimental structure determination methods in a hydrophobic environment. Our results show that adding or removing only one residue at the end of the peptide can totally change its orientation and/or penetration into a lipid environment. However, it does not seem possible to establish a simple rule on this. So if peptides cannot penetrate into a lipid environment, stabilization induced by lipid interaction cannot occur during experimental structure determination methods.

### Evolution of sequences and structures of the IL-2Rγc and IL-2Rβ chain receptors

As we obtained good results for known TMD structures, we used the same method to determine the TMDs of the different sequences of IL-2Rβ and IL-2Rγc for *Homo sapiens, Bos taurus, Mus musculus *and *Rattus norvegicus*.

The IL-2Rγc chain sequence was much more conserved than for IL-2Rβ: this can be explained by the fact that IL-2Rγc is shared by the IL-2 Receptor (IL-2Rβ/IL-2Rγ) and by the IL-4, IL-7, IL-9, IL-15 and IL-21 receptors. The conserved region of IL-2Rγc (v267**G**sM**G**LI273) contains a GG3 pattern, which is important for dimerization and may be involved in the recruitment of other receptor chains. Unexpectedly, mice, rat, dog, cow and pig sequences presented a proline just before this conserved region. This proline is involved in a kink on the extracellular side of the TMD helix, contrary to the case of anthropoids and the duck-billed platypus.

**Figure 6:**
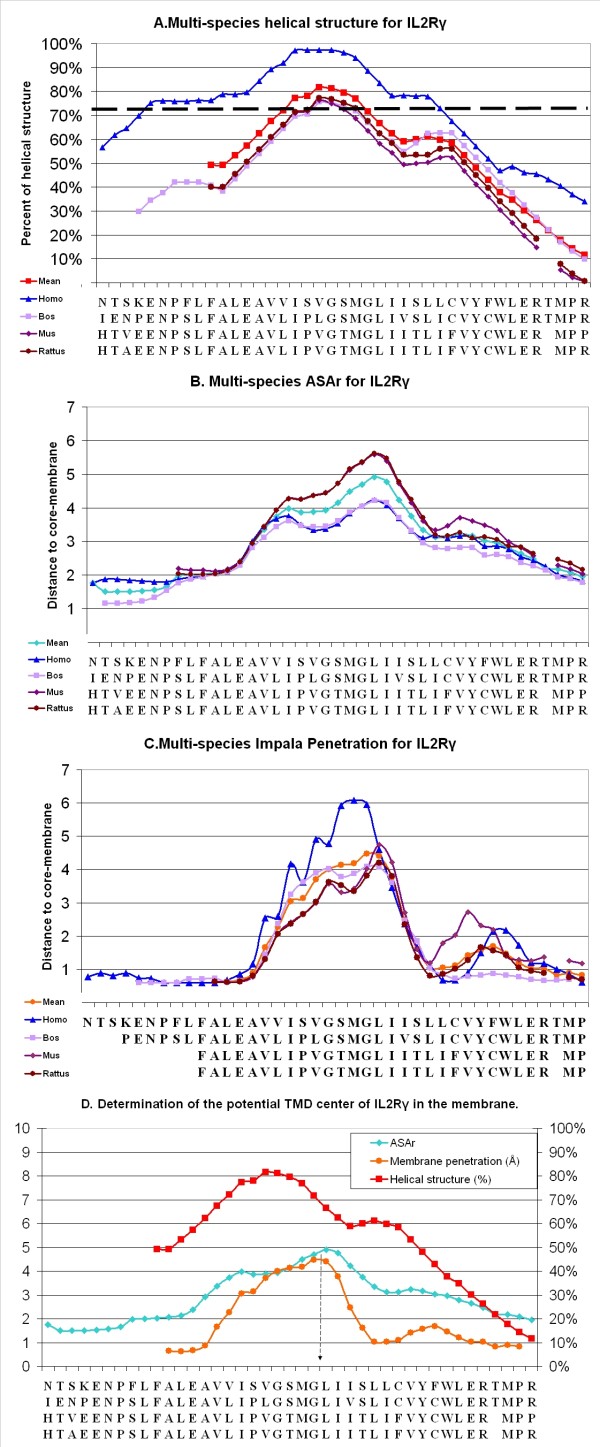
**Determination of the potential TMD center of IL-2Rβ and IL-2Rγ in the membrane**. According to the statistical results from the *PepLook *scan run for the IL-2Rβ **(4) **and the IL-2Rγ **(5), (A) **the average percentage of helical structure in all the structures obtained with a window of 19 a.a. is shown for the different species as follows: *Homo sapiens *(blue), *Bos taurus *(purple), *Mus musculus *(dark purple), and *Rattus norvegicus *(dark red). The amino acid on the × axis represents the center of the considered scan-peptides, according to the species considered. The inter-species mean is shown in red and the acceptable TMD limits are represented by a dashed line at 75%. **(B) **The average ASArs are shown for the 4 species with a window of 5 amino acids; the inter-species mean is shown in blue. **(C) **We show the average depth of the penetration of the 19 a.a. long peptides, with a window of 3, in the membrane from 10 to -10 Å for the 4 species, and the inter-species mean (orange). **(D) **The means of each parameter are shown in the same graph, and the deducible TMD center is indicated by a dashed arrow. (*) Blue stars show the single variations described for *Homo sapiens*.

TMD predictions should be equivalent, since the sequence alignments present conservation of some charged, polar and interfacial residues near the putative TMD. The four species showed similar results (Figures 4 &6) and this similarity allowed us to use inter-species means of each different parameter to avoid isolated ambiguous values: the residue presenting the highest ASAr or best *Impala *insertion values for *Homo sapiens *was characterized by a double maximum (see blue stars in Figure 4-B &4C). The first result was not correct according to the charged residues, which were then inside the membrane. Using results across species showed a better correlation (Figure 6-B). Moreover, ASA values were more precise here than values from 2D methods (because we calculated them on 3D-structures and not with average values) and we found *Impala *to be a good complementary parameter where two close maxima were observed, as in the hIL-2Rβ case.

Finally, we found that the TMDs of IL-2Rβ and IL-2Rγ were respectively centered between F255-G256 and G271-L272. The delimitation of the TMD depends on the tilt angle, which was lower than 30°, so TMDs with interfaces are to be limited to W243-C267 for IL-2Rβ and F258-L283 for IL-2Rγ.

Another advantage of this method is the possibility of calculating MFP percentage levels [22]: our results showed that these were lower than 40% for the I258-N269 segment from IL-2Rβ, in contrast to the remainder of the sequence; so this domain needs interactions with another molecule to stabilize its structure. According to this MFP analysis, I241 Y262 and I265 residues from IL-2Rβ could therefore interact in particular with lipids or proteins which could stabilize the structure of the chain receptors. Similar results were found for IL-2Rγ: residues in L272-I274 and in C278-W282 presented MFP percentages of between 40 and 30% and may interact with the cytoplasmic part of IL-2Rβ and/or lipids. The interfacial residue N254 presented a very low MFP percentage (14%) and may be important for interaction.

The extremities of the IL-2Rβ and IL-2Rγ TMDs present a very high level of conservation and may be involved in a stable orientation in every species. These residues are implicated in interactions with lipids or in the recruitment of several chains known to be co-localized with IL-2Rγc: IL-2Rβ, IL-2Rα, IL-15Rα and IL-7Rα[8-24]. In addition, Constantinescu[25] has described an essential hydrophobic motif for EPO-R activity, at the juxta-membrane side, with conserved hydrophobic residues in many cytokine receptors located in front of functional domains (such as box-1). Homologs of this interfacial motif are found in both cytoplasmic sides of TMDs, presenting a low MFP, and may modulate protein orientation: LxxxFW for IL-2Rγ and LxxxLI for IL-2Rβ.

## Conclusions

In conclusion, our scan-based method is more accurate than 2D methods for the delimitation of TMDs, especially for polymorphic domains or across different species. We validated our approach with structures whose NMR-structures are known and applied it to IL-2Rβ and IL-2Rγc. These scans also allowed us to obtain more information about the structure and polymorphism of TMDs, about MFP and potential interactions, and about residues that are important in membrane insertion. A webserver for this *Peplook*-based method was developed during this study and a *Peplook *webserver will be accessible in a few months at http://www.fsagx.ac.be/bp.

The method described in this paper is currently being developed to model longer target sequences of 34 residues. Preliminary results correlate our predictions of TMD limits with the *Impala *method and suggest that polymorphism is also better identified for those sequences in a rational calculation time. Moreover, this method will describe how already folded stable domains can be involved in the folding of flexible domains, such as the domain of IL-2Rβ.

## Methods

### Sequence and inter-species alignments

We used reference sequences for both proteins, IL-2Rβ (*Homo sapiens *gi:4504665, *Pan troglodytes *gi:78486576, *Macaca mulatta *gi:109094076, *Canis familiaris *gi:73969771, *Bos Taurus *gi:119894052, *Rattus norvegicus *gi:6981098, *Mus musculus *gi:6680427, *Equus caballus *gi:149743257 and *Ornythoryncus a*. gi:149430125) and IL-2Rγc (*Homo s*. gi:4557882, *Pan t*. gi:114689013, *Macaca m*. gi:78486582, *Canis f*. gi:50978962, *Bos t*. gi:27819628, *Rattus n*. gi: 61097900, *Mus m*. gi:7305181, *Callithris j*. gi:146199402 and *Sus scrofa *gi:148747300).

Sequences were aligned for each protein using Clustal-W http://www.ncbi.nlm.nih.gov/tools/cobalt and we modeled proteins from each species whose IL-2Rβ and IL-2Rγ sequences were both available.

### TMD delimitation using 2D methods

We used 2D-methods evaluated by Punta[14] and Zhou[15]: TMHMM2 http://www.cbs.dtu.dk/services/TMHMM, split4 http://split.pmfst.hr/split/4/, THUMBU http://www.smbs.buffalo.edu/phys_bio/Softwares-Services_files/thumbup.htm, Sosui http://bp.nuap.nagoya-u.ac.jp/sosui/, HMMTOP http://www.enzim.hu/hmmtop/html/submit.html and memsat3 http://bioinf.cs.ucl.ac.uk/psipred/.

### The *PepLook *procedure[16]

The *PepLook *method uses a *de novo *search for energy minima through an iterative *in silico *Boltzman-stochastic procedure. For each sequence, *PepLook *generates 1 to 5 million structures: each structure is randomly created by attributing to each amino acid from the sequence, a couple among the set of 64 phi/psi couples, described by Etchebest[26] as being sufficient to describe almost all protein structures in the protein databank (PDB). The energy of the structures is calculated using force fields as described below and ranked for the 104 structures randomly created during a step. At the end of each step, probabilities for each of the 64 phi/psi couples are modulated according to the energy of the structure: the probability of the first 100 phi/psi couples only found in the energetically favorable structures are increased for the next step, and the probability of the first 100 couples, associated only with energetically unfavorable structures, is decreased. The stabilization of probabilities occurs after a number of steps equal to the sequence length multiplied by 10. During the procedure, structures with the lowest energy are retained so as to obtain the 99 best models at the end of the procedure; the prime model is the best structure among the 99.

The 99 structures are clustered according to a RMSD (root mean square deviation) for all atoms below 1 Å. In order to run the *PepLook *procedure in the membrane environment, the *Impala *restraints are added to the total energy (see below).

### The *PepLook *scan followed by the *Impala *method (Figure 2)

We scanned target sequences using the *PepLook *procedure[16,17] in a hydrophobic environment with a window of 19 amino acids and a shift of one amino acid (Figure 2A). This window length was selected to be the largest with the best peptide stability, that is to say the lowest number of clusters and the maximum number of structures by cluster (see results). With these scanned peptides, we performed a statistical analysis for each residue, by excluding the 4 amino acids at each extremity of the scanned peptides, and calculating the average of 3 parameters (Figure 2A): we analyzed the structure, the ratio of hydrophobic to hydrophilic accessible surface area (ASAr), and the insertion into an implicit membrane:

The Pex definition[27,28] was used to establish the secondary structure of residues, according to the phi/psi dihedral angles: helical conformation was attributed when the phi/psi dihedral angles couple was in a circle of 80° around the point (phi = 57°; psi = 47°) of the ramachadran plot, and when the distance between (N-)Hi from the backbone of residue i and either the backbone (C=)Oi+4 for the alpha helix, or the backbone (C=)Oi+3 for the 3-10 helix, was lower than 0.3 nm. Beta-sheet conformation of the residue was considered when the phi/psi couple was in a circle of 90° around the point (phi = -129°; psi = 123°) of the Ramachadran plot. In other cases, residues were considered as random coiled.

Hydrophobic and hydrophilic accessible surface areas[29], calculated using 3D-structures via *PepLook*, allowed us to determine the average ASAr for each residue.

Each scanned peptide was then submitted to *Impala *(Figure 2B) in the membrane core between 10Å and -10Å (because of the length of peptide): we calculated the mean of the best z coordinate for each of the 99 structures of a scan from the same window. The best z penetrating value for each central a.a. of peptides was determined with a window of 7 scanned peptides.

We considered that the central residue in the membrane from the scanned sequence was the residue with the highest ASAr and the highest membrane penetration (minimal z). We also verified that the secondary structure for the domain of 18 residues centered on this amino acid was helical, in order to allow its membrane localization.

### *PepLook *energy field calculations

*PepLook *calculates the energy of peptides through an all atom description of structures using the following energy terms:

London - van der Waals energy describes the energy of interaction between unbound atoms, and its contribution was calculated here using the 6-12 Lenard-Jones potential:

where dij is the distance between atoms *i *and *j*, and A and B are coefficients assigned to atom pairs.

The energy of electrostatic interactions between unbound charged atoms was given by Coulomb's equation:

where dij is the distance between atoms *i *and *j*, qi and qj are the respective fractions of unit charges (using FCPAC partial atomic charges[30]). Epsilon(z) is the dielectric constant of the medium whose variation, with the distance z from the membrane center, and this was simulated here between 0.2 and 1 nm by a sigmoid variation (of factor 1) from 1 to 80 C².J-1.m-1, as described by Smith[31].

Intramolecular hydrophobicity energy decreases exponentially with the distance between atoms and was evaluated by the following equation:

where deltaij is equal to -1 if the atoms are both hydrophobic or both hydrophilic, and +1 otherwise; Etri and Etrj are the transfer energy for these atoms (categorized according to seven atomic types[32]) from a hydrophobic phase to a hydrophilic one; fij is the surface ratio of atom i covered by atom j, and fji the surface ratio of atom j covered by atom i; r^0^i is the van der Waals radius of atom i; dij the distance between atoms i and j; rsol is the radius of a water molecule[33].

The solvent contribution in a hydrophilic environment was calculated implicitly by the external hydrophobicity for each atom:

where Etri is the transfer energy and Si is the solvent accessible surface of atom i calculated with a surface precision of 162 points according to the method of Shrake and Rupley[34].

In a membrane environment, we used the *Impala *method and this solvent contribution was replaced by the empirical equation describing the membrane potential energy (see below).

### *Impala *energy fields[18]

The DPPC membrane was implicitly described by an interface function C(z), described previously: in a bilayer, C varies along the normal to the bilayer surface, the z-axis, where z = 0 at the center of the membrane:

where z0 and alpha are determined so that C(z) is equal to 1 if |z| > 1.8 nm, and equal to 0 if |z| < 1.35 nm, as tested for peptide insertion into a bilayer[18].

The interaction of proteins with the membrane was described by the membrane potential energy corresponding to the sum of the membranous hydrophobicity energy (corresponding also to the interface restraint) and of the membrane lipid perturbation due to the peptide insertion:

Where:

where Si is the accessible surface of atom i to solvent, calculated using the method of Shrake and Rupley[34], Etri is the transfer energy by unit of accessible surface area (defined for seven atomic types) for atom i, and zi the position on the z-axis of the projection of the center from atom i. This equation for Epho_mem is the opposite of the Delta-Gtransfer used by the *OPM *method[20] where alpha = 1/0.9Å-1.

where alip is an empirical constant (fixed to 0.018), which tends to increase if interactions are not as favorable as lipid/lipid interactions.

The *Impala *method consists of moving the protein across the z axis orthogonal to the implicit membrane surface with a constant step (here, 0.01 nm). At each step, the energy is calculated for different orientations of the peptide (here, 10000 steps and a maximum orientation of 360°). The best z position and the best insertion angle correspond to the position with the lowest Epot_mem.

### Mean Force Potential (MFP)

The 494 structures optimized by the Richardson group[35] were used by our laboratory to calibrate an energy function and define the mean MFP by residue. This allows the calculation for each residue of the percentage of the mean MFP defined for this residue[22]: the higher the MFP percentage, the closer the atomic environment of the considered residue to the stable atomic environment found in PDB reference structures. Inversely, a low MFP percentage means that the atomic environment is not complete and that atomic energy loss needs to be counterbalanced either by secondary structure stabilization, or by an inter-molecular interaction inducing structural stabilization.

Calculations were made using a quadri-core PC at Hz and 4 Go of RAM.

## Abbreviations

(TMD): Transmembrane domain; (GpA): Glycophorin A; (MFP): Mean force potential; (DPPC): 1, 2-dipalmytoyl-sn-glycero-3-Phosphocholine; (IL): Interleukin; (ASA): Accessible surface area; (ASAr): Hydrophobic to hydrophilic ASA ratio; (RMSD): Root mean square deviation

## Authors' contributions

YC conceived the method and performed acquisition, analysis and interpretation of the data. RB carried out final approval of the method and of the version to be published. LL was involved in critically revising the manuscript. All authors read and approved the final manuscript.

## Authors' information

RB and LL are Research Director and Senior Research Associate at the National Funds for Scientific Research of Belgium (FNRS).

## Supplementary Material

Additional file 1 **Insertion into the membrane of peptides corresponding to the splitting of GpA NMR-structures**. The Gpa NMR structures (1afo) were split into peptides of different length and inserted into an implicit membrane using the *Impala *method: the histogram shows the tilt angle toward the normal to the membrane for inserted peptides, and lozenges correspond to the distance from the center of the membrane. Click here for file
