# Supplementary figures and images for: A new in-silico method for determination of helical transmembrane domains based on the PepLook scan: application to IL-2Rβ and IL-2Rγc receptor chains
Source: BMC Struct Biol. 2011 May 24;11:26. doi: 10.1186/1472-6807-11-26 (PMC3123172; doi:10.1186/1472-6807-11-26)

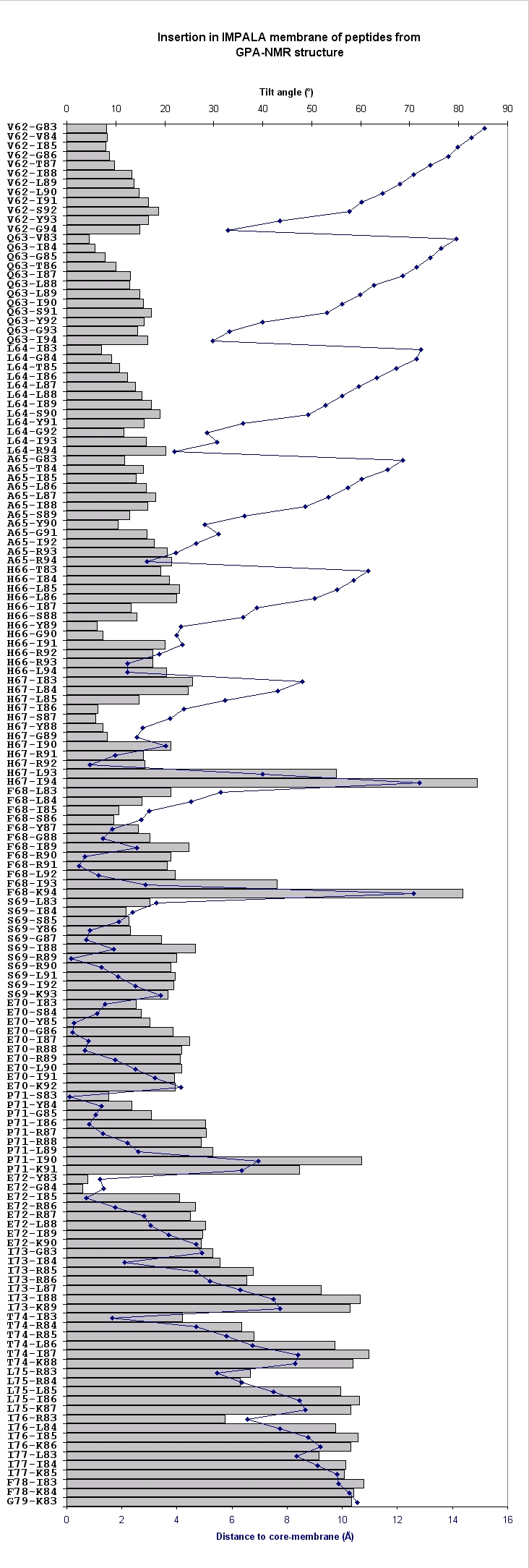

Supplement: Additional file 1 — Insertion into the membrane of peptides corresponding to the splitting of GpA NMR-structures. The Gpa NMR structures (1afo) were split into peptides of different length and inserted into an implicit membrane using the Impala method: the histogram shows the tilt angle toward the normal to the membrane for inserted peptides, and lozenges correspond to the distance from the center of the membrane. [file 1472-6807-11-26-S1.JPEG]
